# Lessons learned from bovine subclinical endometritis: a systematic review exploring its potential relevance to chronic endometritis in women

**DOI:** 10.1530/RAF-23-0035

**Published:** 2024-06-05

**Authors:** Kaltrina Krasniqi, Naomi Black, Erin J Williams, Osvaldo Bogado Pascottini, Sarah Thornton, Siobhan Quenby, Joshua Odendaal

**Affiliations:** 1 Division of Biomedical Sciences, Warwick Medical School, University of Warwick, Coventry, UK; 2 University Hospitals Coventry and Warwickshire, Coventry, UK; 3 MRC Centre for Reproductive Health & The Roslin Institute, University of Edinburgh, College of Medicine and Veterinary Medicine, Edinburgh, UK; 4 Department of Internal Medicine, Reproduction and Population Medicine, Faculty of Veterinary Medicine, Ghent University, Merelbeke, Belgium; 5 Hook Norton Veterinary Group, White Hill Surgery, Hook Norton, UK

**Keywords:** chronic endometritis, subclinical endometritis, cytological endometritis, one health, gynaecology, bovine, dairy cow, pathophysiology, diagnostic techniques

## Abstract

Chronic endometritis (CE) in humans is asymptomatic inflammation of the endometrium, associated with poor reproductive outcomes. Similarly, asymptomatic endometrial inflammation in cows, termed subclinical endometritis (SCE), is associated with adverse reproductive outcomes. While the pathophysiology and treatment options for CE in humans remain poorly defined, the well-known financial implications of SCE in dairy cows have attracted intensive research. We performed a systematic review to determine potential areas of interest in human CE research, by analysing emergent themes that arose in studies of SCE in cows. A literature search for studies of subclinical endometritis in cows published between 1990 and November 2021 was performed across Embase, Medline, Scopus and CINAHL. Studies of symptomatic or clinical endometritis were excluded. A thematic analysis across two broad themes was explored: i) diagnostic methods of SCE and ii) pathophysiology of SCE. In total, 51 bovine studies were included. Twelve studies reported on diagnostic methodology. The primary emergent theme was the use of cytology for the diagnosis of SCE. Cytological analysis has a lower sensitivity than histopathology but is less invasive and more specific than alternative techniques such as ultrasound, vaginoscopy or metabolic markers. The subthemes related to pathophysiology were identified as the type of endometritis, metabolic stress, artificial insemination, infective causes and altered cellular pathways. Despite the lack of symptoms, cellular pathways of inflammation including NF-κB, MAPK and inflammasomes were found to be activated. The key themes related to the diagnosis and pathophysiology of SCE in cows identified in this systematic review highlight potential areas for future research into human CE.

**Lay summary:**

Long-term or chronic endometritis (CE) is a condition associated with inflammation in the womb lining. People with CE commonly experience recurrent miscarriages and subfertility. The cause of CE is poorly understood, and as such there are no specific treatments. Comparatively, a form of CE (i.e. subclinical endometritis (SCE)) has been extensively researched in dairy cows due to its financial impact. A systematic review of cow studies was carried out and key themes around cause and diagnosis of SCE in cows were identified and analysed. In total, 44 studies were included. Six main themes around the way SCE is diagnosed were found, and examining cells (i.e. cytology) was found to be more sensitive and practical than other techniques. Six themes were also found for causes of SCE, notably difficult delivery, metabolic stress and infection. This study shows potential areas for future research into human SCE and provides insight into the causes of disease within humans.

## Introduction

Chronic endometritis (CE) in humans is a condition characterised by asymptomatic inflammation of the endometrium. The normal endometrium has variations in immune cell quantities depending on cyclical changes (Kitaya *et al.* 2016). However, in CE, there are alterations in endometrial cytokine production, damage to endometrial function and abnormal patterns of lymphocyte subsets in the endometrium (Kimura *et al.* 2019, Xu *et al.* 2020). CE is associated with subfertility and reproductive complications such as recurrent implantation failure (RIF) and recurrent pregnancy losses (RPLs) (Li *et al.* 2020). A uniform or globally accepted diagnostic criterion for human CE is not available, but it is commonly diagnosed by immunohistochemical staining of CD138 expressing plasma cells within the endometrium (Puente *et al.* 2020). While CE presents a potential treatable cause of reproductive failure in humans, its pathophysiology and treatment are still not fully understood (McQueen *et al.* 2014).

Conversely, inflammation of the bovine endometrium has been extensively explored due to its well-established negative economic impact on the dairy farming industry and animal welfare (Lee & Kim 2007). Cows can present with several different forms of endometritis. Clinical endometritis is characterised by uterine pus and presents with purulent or mucopurulent uterine discharge accompanied by endometrial inflammation often diagnosed via endometrial cytology (Sheldon 2020). Subclinical endometritis (SCE) is characterised by asymptomatic inflammation of the endometrium, much like human CE, with an increased proportion of polymorphonuclear (PMN) cells, often diagnosed via endometrial cytology (Sheldon *et al.* 2019). This subset may also be referred to as cytological endometritis. For the purposes of this review SCE refers to both SCE and cytological endometritis. SCE is the cause of infertility in an estimated 15–67% of dairy cows, and each case causes significant financial burden due to the costs of treatment, impaired reproductive performance and production losses (Mahnani *et al.* 2015). Research into dairy cow SCE has highlighted a clear pathophysiology in comparison to human CE, with risk factors, potential infective causes and clinical consequences being better understood than in the human. A diagnosis is carried out via a range of methods, including cytology, vaginoscopy, transrectal palpation, ultrasonography of the reproductive tract and biopsy of the endometrium (Wagener *et al.* 2017).

Anatomically, the human and bovine uteri are structurally similar, consisting of the endometrium, the surrounding myometrium and an external perimetrium of loose connective tissue. The bovine uterus is ‘bicornuate’, with the common uterine body separating into two coiled uterine horns, whereas in humans, the organ is described as ‘simplex’, as it consists of a single common uterine body. At a cellular level, the endometrium in both species consists of a stromal layer that contains glandular tissue, underlying a simple columnar epithelium. In the cow, the endometrium contains caruncles; large, vascularised protrusions that have very little glandular tissue and form the maternal side of the placental connections with the developing embryo (Simões & Stilwell 2021). These are absent in the human. The potential for utilising studies of SCE in the bovine population to further understand human CE has not yet been explored. However, it is increasingly recognised that animal studies provide a wealth of knowledge for translational application in clinical research, with emphasis on the need for systematic reviews of animal studies to provide these new insights (Hooijmans & Ritskes-Hoitinga 2013). This offers an opportunity for the analysis of endometritis through a comparative biology lens using knowledge generated in the dairy cow to inform future pathophysiological studies in humans.

The aim of the present study was to systematically review the current knowledge regarding the diagnosis and understanding of the pathophysiology of subclinical endometritis in cows in order to identify diagnostic and pathophysiological pathways that had not yet been explored in human studies. The application of a thematic analysis provided a framework for interpretation of the bovine literature, from the perspective of a human studies, and provided a basis for directing the focus of future studies of CE in the human. Thus, the present study informs on the development of novel strategies for the diagnosis and management of chronic endometritis in humans.

## Methods

A systematic review with an emergent theme thematic analysis was undertaken. This review was prospectively registered on PROSPERO CRD42022264763.

An electronic systematic search of EMBASE, MEDLINE, Scopus and CINAHL from 1990 until November 2021 was performed of the literature on SCE or cytological endometritis in the cow. The search strategy included MESH headings; endometritis, reproductive health, female infertility, cow, bovine, reproduct*, chronic endometritis and cow*. No limits or filters were used. The search strategy and number of search results are presented in Supplementary material 1 (see section on supplementary materials given at the end of this article). In addition, papers provided by expert authors were also included to further contextualise thematic discussion (EW and OBP).

Eligibility assessment of studies by title and abstract was performed independently by two reviewers (KK and JO), with discrepancies resolved by discussion. We included studies that reported on the pathophysiology, diagnostic techniques and associated factors of SCE in dairy cows. Any studies of non-dairy cows, *ex vivo* or *in vitro* studies, symptomatic endometritis cases, case reports and reviews were excluded. There were no restrictions on farm type, setting or location. Full-text review to confirm eligibility was performed by KK and JO. Data extraction was performed by KK using a bespoke data extraction tool presented in Supplementary material 2, with review by a second reviewer where required (JO).

An emergent framework thematic analysis was performed to identify key themes within the included literature. Papers were analysed according to the main concepts and aims of each study, and this helped us identify the main emergent themes of the included papers. Each paper was then compared against these emergent themes to allow the identification of subthemes and the systematic building of ideas relating to these subthemes. This methodology was chosen to identify themes from cow studies which could be translated to human theories and studies.

## Results

**Figure 1:**
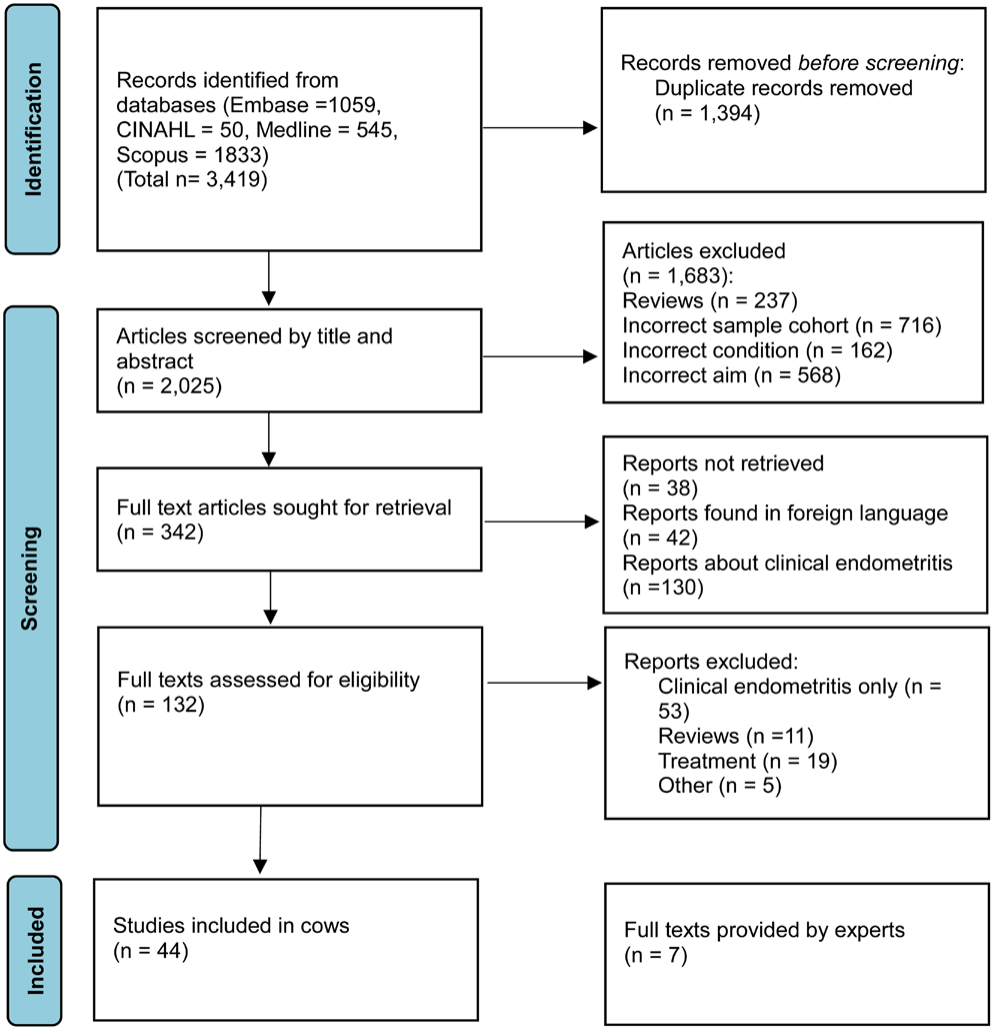
PRISMA diagram for study selection.

The literature search identified 3419 studies, of which 342 studies were eligible for full-text review and 44 studies were identified for further analyses. An additional seven texts identified by expert authors were included. Overall, the review comprised 51 studies. An overview of the selection process is shown in Fig. 1.

Emergent theme analysis revealed two key themes: i) diagnostic methods and ii) pathophysiology. While extensively investigated in studies involving cows, these themes remain inadequately and insufficiently explored in human studies.

## Theme 1: methods for diagnosing SCE in dairy cows

**Figure 2:**
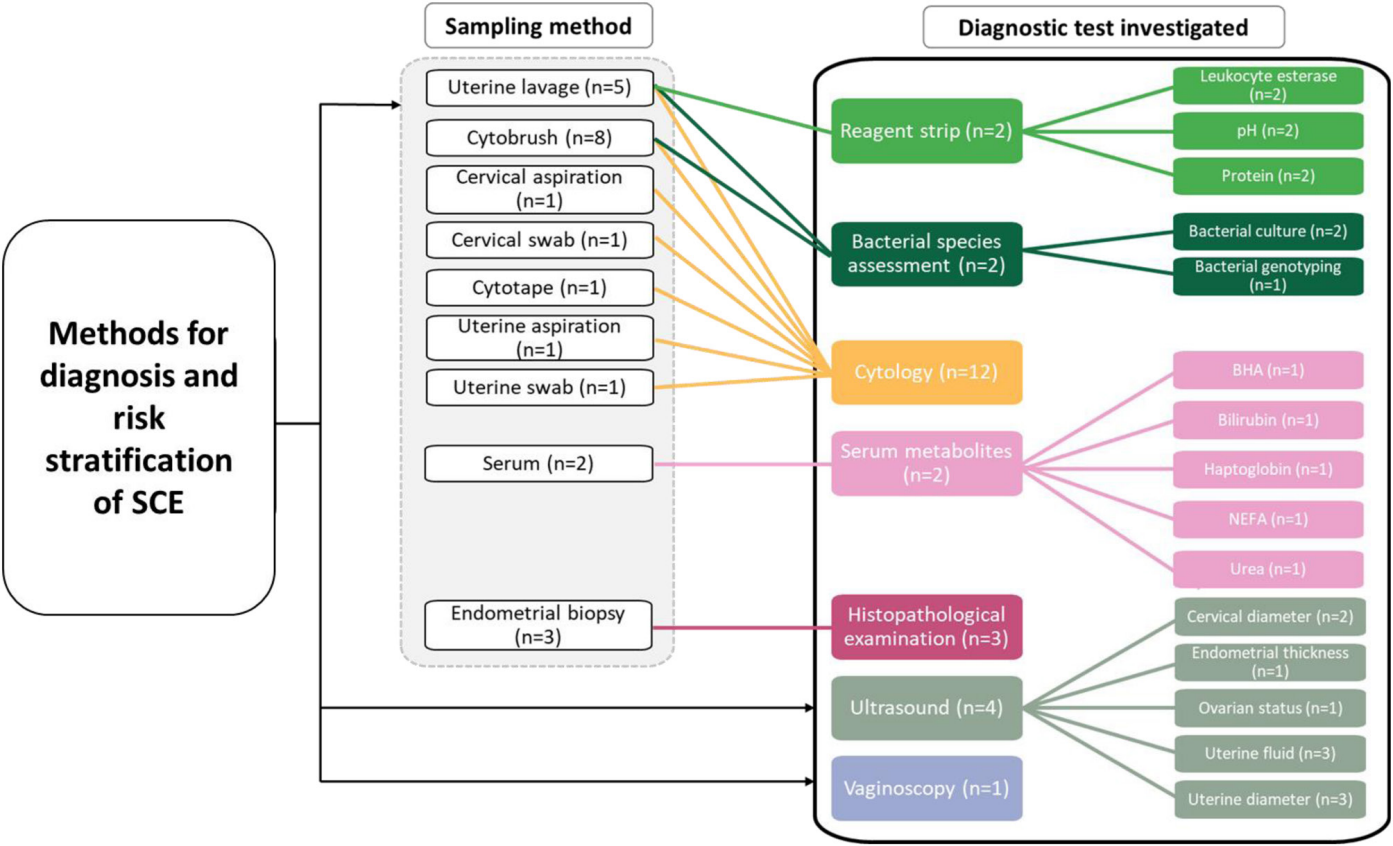
Methods for diagnosis and risk stratification of SCE investigated by the papers included in the study.

Twelve papers explored the optimum method for diagnosis of SCE in dairy cattle (Table 1). Thematic analysis identified six key subthemes in the diagnostic literature: i) cytology for diagnosis, ii) cytological assessment compared to histopathology, iii) cytological assessment compared to alternate diagnostic methods of ultrasound or vaginoscopy, iv–vi) the use of reagent test strips, serum markers and bacterial culture (Fig. 2).

**Table 1:** Overview of included studies investigating different diagnostic methods for subclinical endometritis in cows.

Study	Study population	Condition(s) investigated	Sampling technique	Diagnostic method investigated						
				CT	US	HP	CA	RS	SM	BCG
Ahmadi *et al.* (2005)	131 slaughtered cows	Acute, subacute, chronic EM	Cervical swab; cervical aspiration; uterine swab; uterine aspiration; endometrial biopsy	✓	–	✓	–	–	–	–
Barlund *et al.* (2008)	221 cows 28–41 days PP	CE and SCE	Cytobrush; uterine lavage	✓	✓	–	✓	–	–	–
Pascottini *et al.* (2016*a*)	32 cows pre and post slaughter	SCE	Cytobrush; endometrial biopsy; uterine lavage	✓	–	✓	–	–	–	–
Brodzki *et al.* (2014)	60 cows PP (30 EM, 30 healthy)	SCE	Cytobrush; uterine lavage	✓	✓	–	–	–	–	✓
Cheong *et al.* (2012)	563 cows 40–60 days PP	SCE	Uterine lavage	✓	–	–	–	✓	–	–
Egberts *et al.* (2016)	53 cows 64–1018 days PP	SCE	Cytobrush	✓	–	–	–	–	–	–
Kasimanickam *et al.* (2004)	228 lactating cows	SCE	Cytobrush	✓	✓	–	–	–	–	–
Kaufmann *et al.* (2010)	209 cows up to 35 days PP	CE and SCE	Serum samples for NEFAs, BHA, bilirubin, urea; cytobrush	✓	–	–	–	–	✓	–
Madoz *et al.* (2013)	53 cows 27–56 days PP, and 418 cows 21–62 days PP	SCE	Cytobrush	✓	–	–	–	–	–	–
Meira *et al.* (2012)	76 cows 21–47 days PP	SCE	Cytobrush; endometrial biopsy	✓	✓	✓	–	–	–	–
Nyabinwa *et al.* (2020*a*,*b*)	466 cows 21–60 days PP	CE and SCE	Cytotape	✓	–	–	✓	–	–	–
Ricci *et al.* (2017)	70 healthy cows 30–60 days PP	SCE	Uterine lavage; serum sample for haptoglobin	✓	–	–	–	✓	✓	✓

BCG, bacterial culture or genotyping; CA, clinical assessment (e.g. external inspection, vaginoscopy); CE, clinical endometritis; CT, cytology; EM, endometritis; HP, histopathology; PP, post partum; RS, reagent strips; SCE, subclinical endometritis; SM, serum metabolites; US, ultrasound.

### Subtheme 1: cytological assessment for the diagnosis of SCE in dairy cows

A strong emergent theme was the use of PMN quantification through cytological assessment of the endometrium for diagnosis of SCE in cows (Fig. 2).

In practice, this method generally involves passing a cytobrush through the cervix and into the uterus, and by administering gentle pressure per rectum, the operator ensures the brush makes contact with the endometrium and then gently rotates the instrument to ensure a good cell sample is obtained (Kasimanickam *et al.* 2006, Rana *et al.* 2020).

### Cytobrush

An endometrial cell sample can also be collected for cytological assessment by uterine lavage, or by cytotape – an adaptation of the cytobrush technique where rolled surgical tape collects the endometrial cells instead of a brush (Pascottini *et al.* 2016*b*). Sampling can be performed faster and easier via the cytobrush (or cytotape) method compared to uterine lavage (Brodzki *et al.* 2014).

Both cytobrush and uterine lavage provide samples of similar quality and cellularity, with lavage achieving a 14.3% sensitivity and 84% specificity for the diagnosis of SCE (Barlund *et al.* 2008, Brodzki *et al.* 2014).

The timing and location of cytological sampling are key in ensuring an accurate representation of the endometrium. It has been suggested that cytobrush analysis may require at least two samples from different locations, preferably the uterine horns, to be able to effectively represent the PMN cell levels, although currently a single-site approach remains the standard (Egberts *et al.* 2016). There is a risk of a high false-positive rate if the cytological sample is collected too early in the oestrous cycle, due to normal infiltration of PMN cells (Egberts *et al.* 2016). Following calving, physiological inflammation of the endometrium occurs in order to facilitate uterine involution and endometrial repair, so the level of PMN infiltration that signifies SCE differs across the postpartum period (Foley *et al.* 2015). This is presently overcome by standard practice involving sampling >20 days post partum.

### Subtheme 2: cytology vs biopsy

Biopsy or histopathology was discussed in three studies and was suggested as the definitive diagnostic tool to diagnose SCE with greater sensitivity than cytology (Barlund *et al.* 2008). Compared to cytology, histopathology is regarded as a more sensitive diagnostic tool (Pascottini *et al.* 2016*a*) and is therefore considered the most definitive method for identifying tissue inflammation and endometrial lesions (Meira *et al.* 2012). However, biopsy is comparatively expensive, time-consuming and has negative implications on subsequent fertility outcomes, which could affect the interpretation of results or effectiveness of treatments tested (Barlund *et al.* 2008, Pascottini *et al.* 2016*a*). Thus, using cytological assessments has proven to be less invasive and fairly precise (Barlund *et al.* 2008).

### Subtheme 3: cytology vs ultrasound or vaginoscopy

Seven studies used cytological analysis of the endometrium as a baseline for comparison with alternate diagnostic methods. One of the included articles compared a cytobrush technique to ultrasound and vaginoscopy. Barlund *et al.* (2008) hypothesised that assessment of intrauterine fluid accumulation may improve the diagnostic capability of ultrasound for SCE. They found that sonographic uterine fluid assessment had poor sensitivity but good specificity for the diagnosis of subclinical endometritis, especially in cases of increasing volumes of intrauterine fluid. Vaginoscopy proved to be rapid and simple but had only moderate agreement with the cytobrush results and required exudate analysis to make a positive diagnosis. This suggests ultrasound and vaginoscopy are more effective techniques for diagnosis of clinical endometritis, where vaginal discharge is prevalent. Thus, by their very nature, these are not appropriate tests for the determination of subclinical endometritis.

### Subtheme 4: reagent test strips for diagnosis

Reagent strips are a surrogate test and have the potential for significant benefit in the diagnosis of SCE as they could facilitate an on-the-step ‘point-of-care’ diagnosis, negating the time and financial consequences of laboratory analysis. Their use in this context was explored in two studies, and while there was a positive correlation between the test strips and a diagnosis of endometritis, with leukocyte esterase, pH and protein all increased in cows with SCE diagnosed via cytology, reagent strips were considered a poor diagnostic technique due to the low specificity and sensitivity seen across both studies (Cheong *et al.* 2012, Ricci *et al.* 2017).

### Subtheme 5: serum compounds and proteins for assessing the diagnostic risk of SCE

Serum markers to identify risk of SCE were explored in two studies. Urea and non-esterified fatty acids (NEFAs) were investigated as markers of risk for SCE. Urea levels were raised at 1 week post partum (Kaufmann *et al.* 2010). NEFAs have a sensitivity and specificity of 35% and 89%, respectively, for subclinical endometritis (Kaufmann *et al.* 2010), and it has been found that high levels of NEFAs can affect blood PMN functions (Kaufmann *et al.* 2010). Serum haptoglobin, a nonspecific acute phase protein that increases in response to inflammation, was examined as a potential marker by one group. However, no significant difference was found in serum haptoglobin between those with positive or negative cytology for SCE (Ricci *et al.* 2017).

### Subtheme 6: bacterial culture

A final theme that emerged was bacterial culture obtained from a uterine swab sample. While there is consensus that clinical endometritis in cows is the result of bacterial infection, the association between the presence of specific pathogenic bacteria and SCE is not well established (Pascottini *et al.* 2023). Of the included studies, two studies discussed bacterial culture as a diagnostic method for SCE. *Trueperella pyogenes* and *Escherichia coli* were isolated in both studies (Brodzki *et al.* 2014, Ricci *et al.* 2017); however, a direct association between SCE and the bacterial infection has not been confirmed. The current consensus suggests that SCE is the result of a dysfunctional metabolic and inflammatory response rather than an infectious agent (Pascottini *et al.* 2023).

## Theme 2: pathophysiology

**Figure 3:**
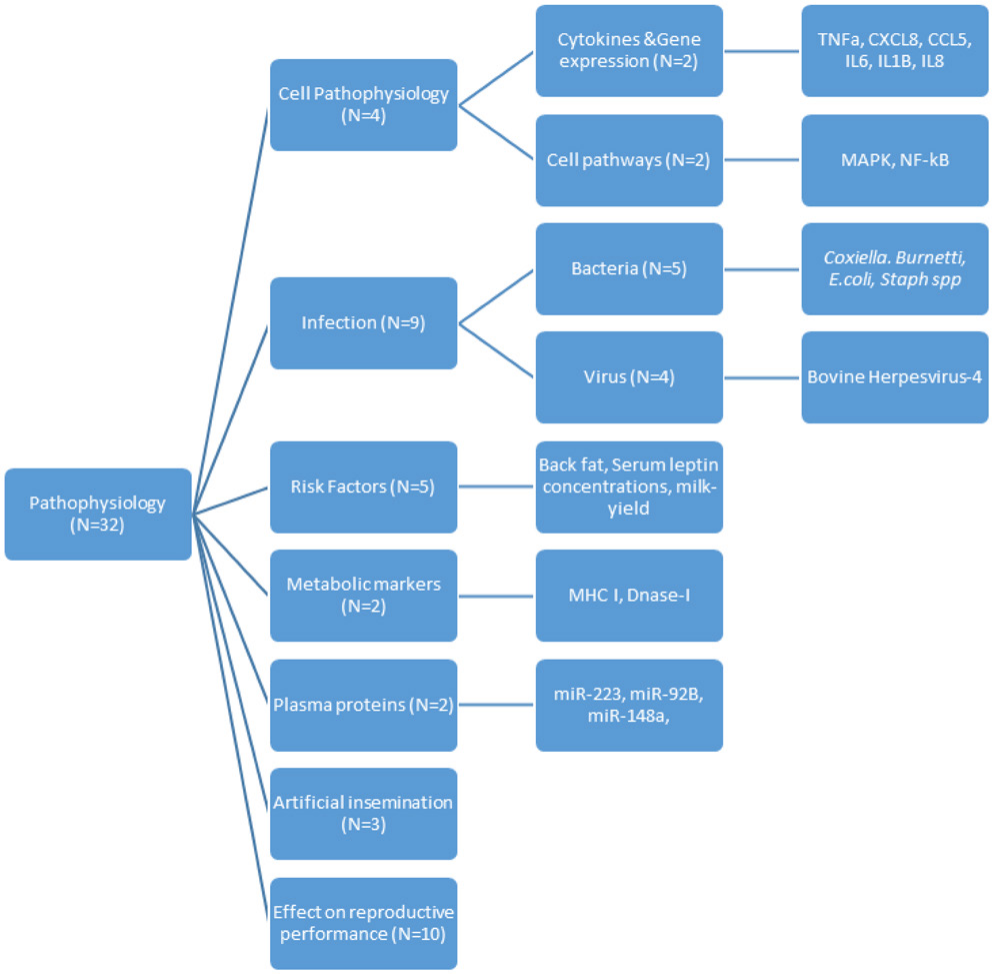
Flow chart of pathophysiology themes and subthemes presented in the papers included in the study.

Pathophysiology was discussed in 33 papers, and data are presented in Table 2. A thematic analysis of the theories surrounding pathophysiology of SCE in cows revealed five key themes: the type of endometritis, metabolic stress, artificial insemination and the postpartum period, and infective causes and cellular pathways. An overview of the identified themes is presented in Fig. 3.

**Table 2:** Overview of included studies investigating the pathophysiology of subclinical endometritis in cows.

Study	Cows, *n*	Type of EM	PMN%	Study aims
Bacha & Regassa (2010)	59	SCE	≥5% neutrophil count	Effect of SCE on reproductive performance
Barański *et al.* (2013)	222	SCE	4–18%	Impact of EM at week 4 and 6 PP
Barrio *et al.* (2015)	94	SCE	≥5% neutrophil count	Effect of SCE on reproductive performance
Pascottini & LeBlanc (2020)	58	SCE	>5% neutrophil count	Metabolic markers
Pascottini *et al.* (2017)	873	CTE	≥1% PMN	Effect of AI on EM status
Pascottini *et al.* (2016*a*,*b*)	512	CTE	≥1% PMN	Success of insemination, conception rates with CYTO and without
Bruun *et al.* (2002)	2144	Metritis	–	Risk factors
Cheong *et al.* (2011)	779	SCE	>10% PMNL	Risk factors: herd size, calving pen, postpartum housing, pen moves, parity, ketosis, milk production
De Biase *et al.* (2018)	37	CE	–	*Coxiella burnetii* detection by PCR
Diaz-Lundahl *et al.* (2021)	1648	CTE	≥3.0% PMN	Effects of AI on fertility
Donofrio *et al.* (2010)	–	^§^	–	Activation of *IL8* gene promoter in BoHV-4 infection of bovine ESCs
Fábián *et al.* (2008)	131	EM	–	Bacterial isolates in endometrial samples
Fagundes *et al.* (2019)	64	SCE	>5% PMN	Expression of *CCL5, CXCL8, IL6, IL1B*genes
Gilbert *et al.* (2005)	141	SCE/CTE	5% neutrophils as cutoff	Prevalence and reproductive performance
Gobikrushanth *et al.* (2016)	15	CTE	>18% PMN^⁋^	Reproductive performance, dry matter intake, milk yield in dairy cattle
Jiang *et al.* (2021)	3	EM	–	Describing the role of miR-92B in inflammatory response in bovine EECs, following LPS stimulation.
Kasimanickam *et al.* (2006)	275*	SCE	–	Effect of clinical/SCE on cow fertility***
Kudo *et al.* (2021)	14	SCE	>1, or a PMN ratio >10%	Microbiota of primiparous cows with and without EM
Lee *et al.* (2018)	407	CTE	14% at 4 weeks PP	Impact on reproductive performance
Miller *et al.* (2019)	112	CTE	>18% PMN 21 DPP	Plasma protein levels
Nyabinwa *et al.* (2020*b*)	436^†^	SCE	≥5% PMN	Impact on reproductive performance
Peter *et al.* (2018)	116	SCE	≥5%^‡^	Monitoring uterine health of cows with* Lactobacillus buchneri*
Plöntzke *et al.* (2010)	201	SCE	≥5% PMN	Prevalence
Salasel *et al.* (2010)	92	SCE	≥3% PMN	Impact and risk factors
Santos & Bicalho (2012)	16	EM	–	Bacteria colonies in uterine fluid
Senosy *et al.* (2011)	198	SCE	>5% PMN	Metabolism investigated in SCE cows
Sens & Heuwieser (2013)	149	SCE	4 to 18% PMN	Bacterial infections’ effects on uterus
Sharma *et al.* (2021)	41	SCE	≥5% PMN	Back fat, serum leptin concentrations, endometrial cytology, milk yield
Vallejo *et al.* (2018)	166	SCE	>5% PMN	Effect of EM on reproductive behaviour
Vieira-Neto *et al.* (2014)	1569	CTE	≥5% PMN	Reproductive performance effect of CTE and anovulation
Yin *et al.* (2019)	3	–	–	MAPK pathway, LPS induction of inflammation and the effect of ferulic acid on EECs
Zhao *et al.* (2018)	10	SCE	–	Activation of NF-κB and promotion of miR-223 in LPS-induced EM

*106 were subclinical; ^†^SCE = 13; ^§^BoHV-4 serum negative, healthy cows at slaughter; ^⁋^From cytobrush samples collected between 21 and 33 DPP or greater than 10% PMN between 34 and 47 DPP; ^‡^Cows were classified as having SCE.

AI, artificial insemination; CM, chronic endometritis; CTE, cytological endometritis; DPP, days post partum; EECs, endometrial epithelial cells; EM, endometriosis; ESCs, endometrial stromal cells; PP, post partum; SCE, subclinical endometritis.

### Subtheme one: type of endometritis

**Figure 4:**
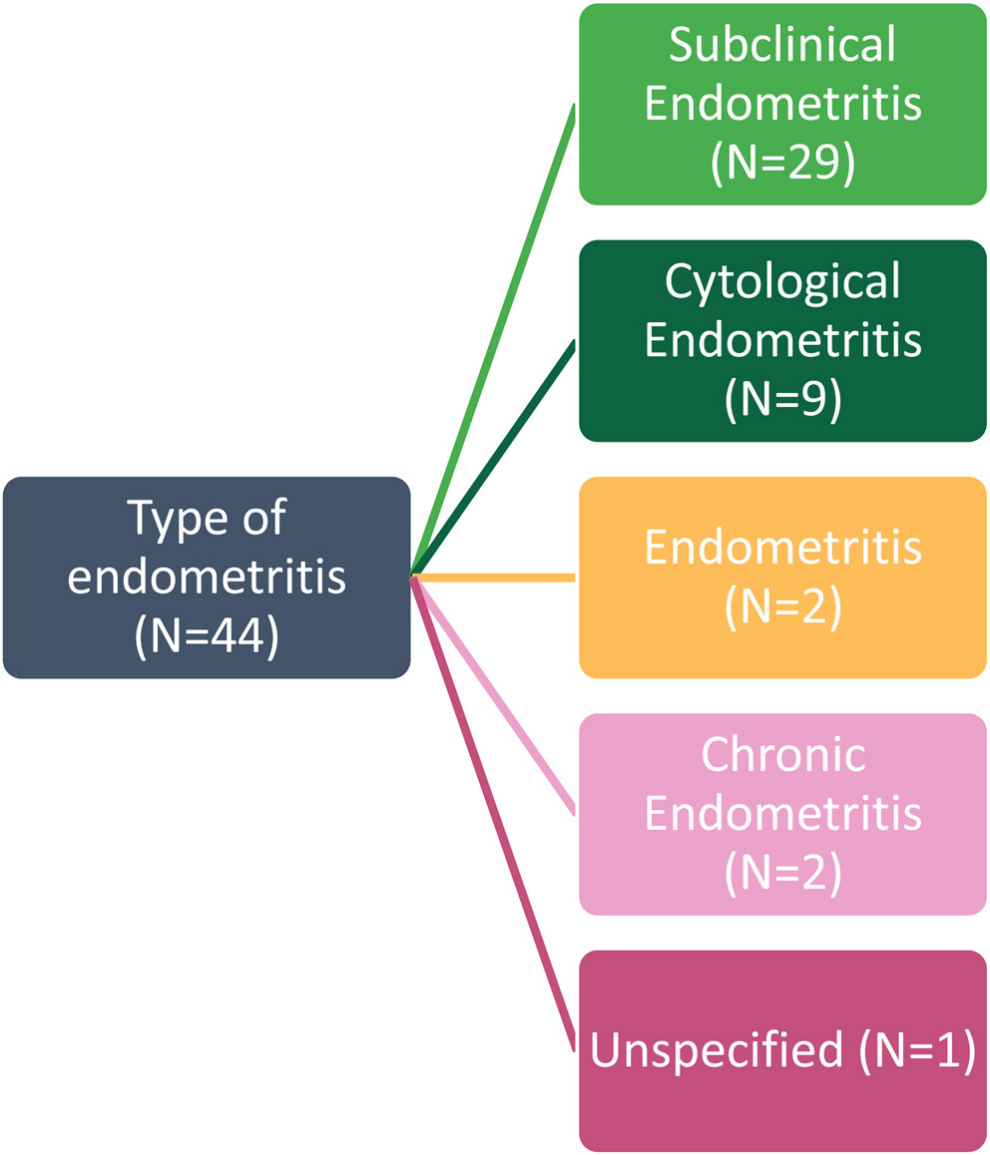
Representation of the breakdown of terms used to describe non-‘clinical endometritis’ in the included studies.

A thematic analysis of the articles highlighted a crucial theme: the terminology and definition of endometritis. The most frequently used terms were SCE and cytological endometritis, used in 29 and 9 of the 44 articles, respectively (Fig. 4). Although different terms were used, the diagnostic criteria were the same; the presence of PMN cells greater than 5% in a field of 300 endometrial cells, 35 days post partum and a lack of clinical signs and symptoms (Wagener *et al.* 2017).

### Subtheme two: metabolic stress

SCE has been commonly viewed to be the result of an underlying dysfunctional metabolic process and has been associated with metabolic risk factors (Dubuc *et al.* 2010 , Pascottini *et al.* 2023). Depletion of energy stores has been postulated to increase the risk of SCE. A number of potential mechanisms have been proposed for this relationship within the literature (Senosy *et al.* 2011, Sharma *et al.* 2021). These stem from the relationship between glucose metabolism and the immune system. Low blood glucose levels in high yielding dairy cows are common due to the redirection of glucose for milk production. As a major fuel for immune cells, low blood glucose can lead to dysfunctional immune function. Blood glucose has been found to be lower in cows affected by SCE than control cows, reinforcing this link (Senosy *et al.* 2011). Furthermore, low back fat thickness (BFT) and body condition scores (BCS) have also been shown as risk factors for SCE. These have both been correlated with low blood glucose (Sharma *et al.* 2021), suggesting a link between SCE and metabolism-related risk.

### Subtheme three: artificial insemination and the postpartum health issues

Artificial Insemination (AI) is extensively used in dairy herds for genetic improvement and economic benefits. It allows farmers to breed their cows with the best sires to produce optimal offspring. AI reduces the risk of infection transmitted through natural mating; however, if it is not carried out properly, there is the risk of introducing debris and bacteria into the cows’ uterus, which may trigger an immune response and subsequent endometritis, which is rare in the cow (Parkinson & Morrell 2019).

In order to maintain optimum milk production, the aim is to get one calf, per cow, per year. With a gestation of around 285 days, a cow would need to be inseminated and become pregnant approximately 80 days after she last calved. This time period is called the calving to conception interval. To achieve a pregnancy by 80 days, a cow needs to be inseminated by around 60 days after calving, this is measured as the calving to first service interval. Health issues that occur in the first 60–80 days after calving can mean a cow is not suitable for insemination, therefore her days to first service, and calving to conception intervals will be prolonged. Both of these measures are an indicator of poor fertility, or poor reproductive health. In addition to metabolic dysfunction, peri- and post-partum risk factors for endometritis include calving related diseases such as retained placenta, dystocia, twin calving, and caesarean section (Potter *et al.* 2010, Lee *et al.* 2018).

It emerged in the thematic review of the bovine literature that the postpartum period is a high-risk period where cows are susceptible to SCE.

A positive endometritis diagnosis correlates with a decrease in the total number of cows pregnant by 300 days post partum (Gilbert *et al.* 2005) and an increase in the calving to first service interval. Calving to conception interval and intercalving periods are significantly higher in cows who have had SCE in the postpartum period (Barański *et al.* 2013, Sharma *et al.* 2021). Cows who experience endometritis also need more inseminations to become pregnant (serves per conception) (Gilbert *et al.* 2005). Thus, the intercalving period is longer due to repeated failed AI attempts. In one study, 16.13% of cows who required more than one service per conception had previously suffered from SCE, whereas only 3.2% of cows pregnant to first service did (Pascottini *et al.* 2016*a*). In another study, the prevalence of cows who had SCE at AI was reported as 25.3% (Pascottini *et al.* 2017).

### Subtheme four: infective causality

#### Subtheme four a: bacterial cause

An important subtheme seen in a number of studies is infiltration of the endometrium with bacteria. Early studies in the cow of the causes of endometritis focused on isolation of microbiota from the uterus of diseased animals (Williams *et al.* 2005, 2007). One body of evidence suggests that endometritis, including SCE was caused by an infection of the endometrium, and several gram-negative bacteria were particularly correlated with disease. Santos & Bicalho (2012) found Fusobacteriaand Proteobacteria were the dominant phyla with Proteobacteria found in 70% of the study population. Brodzki *et al.* (2014) isolated both *E. coli* and *T. pyogenes* in affected groups and Williams *et al.* (2005) defined *E.coli, T.pyogenes*, Fusobacteria and *Prevotella* as ‘uterine pathogens’ due to their consistent isolation from cows with uterine disease. While a number of bacterial species were isolated across different studies, *E. coli* was the most common (Williams *et al.* 2005, 2007, Fábián *et al.* 2008). α-Hemolytic streptococci (AHS) were also isolated in a study conducted by Sens & Heuwieser (2013) in the early postpartum period in cows with a previous diagnosis of SCE. AHS isolation was associated with an increase in PMN cell percentage and a higher risk of SCE at 4 weeks post-partum.

In recent years, the advancement of culture-independent molecular techniques has provided deeper insight into the microbiome of the bovine uterus, and its relationship with endometritis (Santos & Bicalho 2012, Miranda-CasoLuengo *et al.* 2019). Temporal analysis has shown changes in the composition of cows with uterine disease from calving until late postpartum (Santos & Bicalho 2012, Knudsen *et al.* 2016). Loss of bacterial diversity and dominance of the microbiome by a few bacterial taxa were related to the development of clinical disease (Miranda-CasaLuengo *et al.* 2019), and include the common uterine pathogens identified in culture-dependent studies. Studies, however, have reported a characteristic uterine microbiome in cows with cytological endometritis that is largely lacking the common uterine pathogens, and is instead dominated by commensal bacteria, suggesting that they may have a role in inflammatory processes in the endometrium (Wang *et al.* 2018). In contrast, other studies have found no difference in the microbiome between healthy cows and cows with SCE (Pascottini *et al.* 2020). A physiological response to bacterial contamination of the endometrium was proposed by Vieira-Neto *et al.* (2014). The intimate anatomic relationship between the ovarian arteries and uterine veins that is indispensable for the local transfer of prostaglandin from the endometrium to the ovary may also leak bacterial toxins to the developing follicle. These bacterial toxins disrupt the normal secretion of luteinising hormone (LH) and in doing so reduce the size and growth of the first postpartum dominant follicle, therefore impairing its ovulatory capacity. An increase in immune cell infiltration into the surface epithelium, lumen of endometrial glands and surrounding tissue causes occlusion and dilatation of glands. This leads to scar tissue formation in the uterus which is thought to impair implantation of the embryo and maintenance of pregnancy (Vieira-Neto *et al.* 2014).

#### Subtheme four b: viral cause

An additional subtheme identified in the literature is that of viral infection, specifically bovine herpesvirus 4 (BoHV-4). Fábián *et al.* (2008) isolated BoHV-4 in 27/31 SCE cows. One cow had high viral loads in the absence of bacterial infection, however, in the majority of cases pathogenic bacteria were also isolated, suggesting BoHV-4 may not be a lone infectious agent in SCE. Banks *et al.* (2008) have suggested that viruses such as BoHV-4 may lie latent in white blood cells and are reactivated due to peripartum stress and endocrine conditions. It has also been suggested that BoHV-4 may have a positive tropism for endometrial cells (Donofrio *et al.* 2007, Pascottini *et al.* 2016*b*).

#### Subtheme Five: cellular pathways

**Figure 5:**
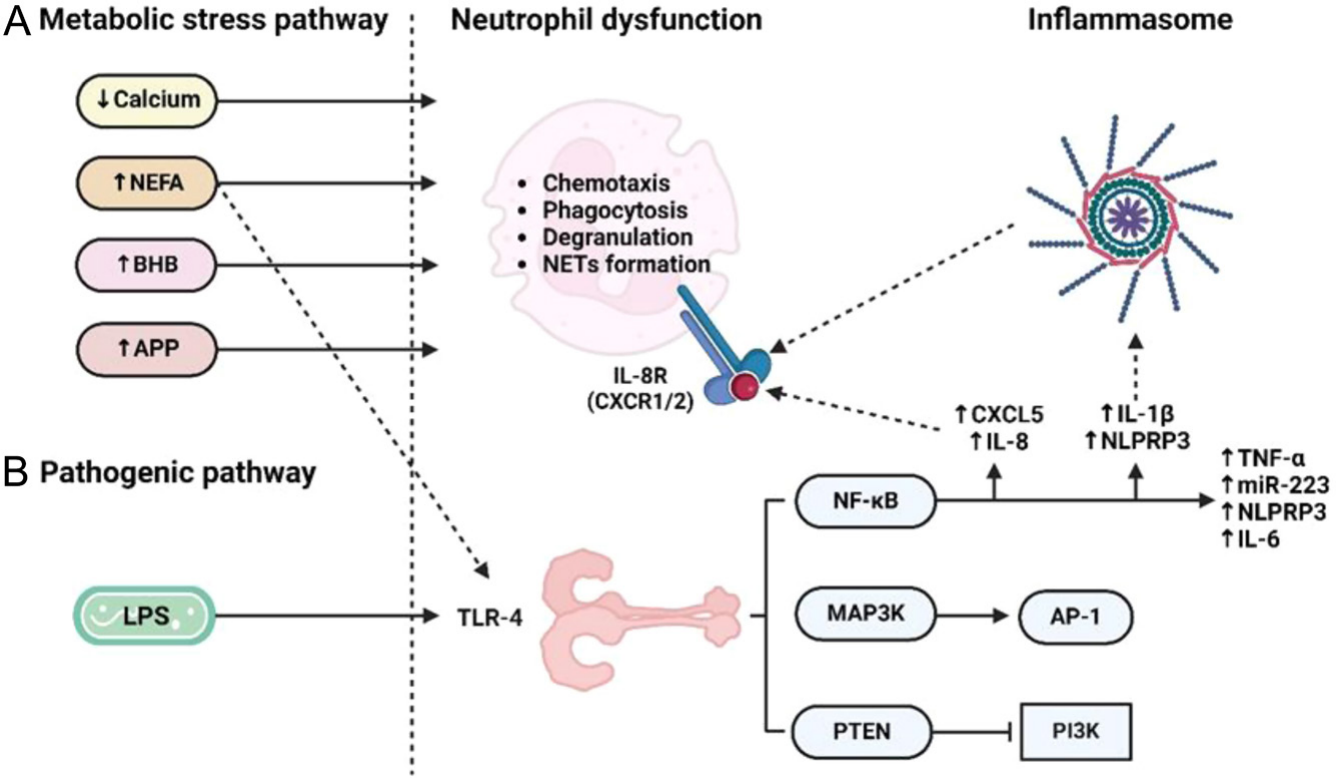
Biomolecular pathways implicated in subclinical endometritis. (A) Metabolic stress: High systemic concentrations of non-esterified fatty acids (NEFAs), β-hydroxybutyrate (BHB), and acute phase proteins (APPs), along with dyscalcemia, contribute to neutrophil dysfunction and persistent endometrial inflammation. NEFAs may additionally activate toll-like receptor 4 (TLR-4). (B) Pathogenic pathway: Activation of TLR-4 by lipopolysaccharide (LPS) triggers: 1) PTEN pathway, promoting cell apoptosis by inhibiting the PI3K pathway; 2) MAP3K cascade, inducing AP-1, a transcription factor linked to apoptosis; 3) NF-κB pathway, leading to pro-inflammatory cytokine release, neutrophil activation via inflammasome formation, and CXCR1/2 activation mediated by CXCL5 and IL-8 (Peter *et al.* 2018, Fagundes *et al.* 2019, Yin *et al.* 2019, Jiang *et al.* 2021, Pascottini *et al.* 2023).

A final subtheme that emerged from our analysis is the cellular pathways that underpin the uterine immune response. These involve the activation of several pro-inflammatory pathways following activation of toll-like receptors on the endometrial cell surface, which trigger downstream inflammatory components including NF-κB, MAPK and the inflammasome. These are summarised in Fig. 5.

## Discussion

This systematic review with thematic analysis of 51 studies investigated the themes surrounding diagnostic methods and pathophysiology of SCE in dairy cows with the aim of identifying under-explored areas in the human condition, CE. The key themes that emerged within the literature were diagnosis and the pathophysiology of SCE. Within these themes, several subthemes were identified including, the significance of endometrial immune cells in aiding diagnosis and the influence of the uterine bacteria in the sequalae of disease. These themes present future potential avenues for research exploration in humans.

A key theme that emerged was the use of cytology as a diagnostic tool. Cytology for CE has not been thoroughly explored in human literature; the current gold standard for diagnosis in humans is histological biopsy (Hartman *et al.* 2011, Abdelazim *et al.* 2015). Cytological endometrial sampling has been shown to reduce patient discomfort in humans in comparison to endometrial biopsy and thus may be a less invasive diagnostic approach for CE in humans (Williams *et al.* 2008, Kumar 2017).

This present study highlights the importance of analysis of immune cells including PMN cells for the diagnosis of SCE in cows. PMN cells are activated in SCE in cows through a number of cellular pathways and result in further activation of neutrophils and other leukocytes (Peter *et al.* 2018, Fagundes *et al.* 2019, Yin *et al.* 2019). Diagnostic criteria for SCE centres on determining the proportion of PMN cells within the endometrial cell population, with specified levels indicating SCE at different time points after calving (Kasimanickam *et al.* 2004). Currently, there is no consensus regarding the diagnostic criteria for CE in humans (Liu *et al.* 2018) and cellular analysis does not focus on PMN numbers. The majority of human studies diagnose CE based on the quantification of plasma cells within the endometrial stroma (Odendaal & Quenby 2021). The lack of a consensus regarding the diagnostic criteria, however, affects the prognostic ability of the test, impeding research in the area (Liu *et al.* 2018). The analysis of alternative immune cell populations, including PMN cells, in the human endometrium offers the potential for a diagnostic alternative or adjunct to current practice.

As well as diagnosis of SCE, pathophysiology emerged as a key theme in the present study. Subthemes of importance included the role of the endometrial microbiome in influencing disease, the cellular response to bacteria, and the impact of AI.

The isolation of gram negative bacteria from the uterus of cows that go on to receive a diagnosis of SCE was a subtheme explored across several of the studies. The predominant bacteria associated with disease were the ‘uterine pathogens’ *E coli, T pyogenes*, *Fusobacteria* and *Prevotella* (Gilbert *et al.* 2005, Williams *et al.* 2005), with proposed bacterial sources including *Fusobacteria, Proteobacteria*, *E. coli*, and AHS. Isolation of AHS was associated with an increased PMN cell percentage and increased risk of SCE at 4 weeks post-partum in cows (Sens & Heuwieser 2013). Gram negative bacteria, specifically *E. coli*, has been implicated in the development of CE in humans. Cicinelli *et al.* (2009) isolated *E. coli* in 11% of women with human CE in an infertility cohort. Indeed, the cell wall endotoxin produced by *E coli*, lipopolysaccharide (LPS) is known to induce the upregulation of plasma cells following infection with *E. coli* in humans (Kimura *et al.* 2019, Sola-Leyva *et al.* 2021). In the cow, LPS is known to induce endometrial inflammation by binding to TLR4 on the surface of endometrial cells and initiating a downstream cascade of inflammatory mediators (Jiang *et al.* 2021). Thus, LPS induced inflammation is likely implicated in human CE but this remains to be explored.

Potentiation of the development of SCE in cows by viral BoHV-4 emerged as a subtheme within the literature. It is suggested that BoHV-4 lies latent in white blood cells and upon changes in the endometrial environment and metabolic status of the cow, BoHV-4 reactivates (Donofrio *et al.* 2010). Investigation of the role of viruses in human CE is lacking, therefore the present study suggests this is an area that merits exploration.

Cellular pathways, including NF-κB and MAPK, are a significant subtheme that has been implicated in the pathophysiology of SCE in cows. miRNA (miR-92b and miR-223) activation from these pathways suggests evidence of chronic disease (Tahamtan *et al.* 2018). The activation and effect of these pathways are underexplored within the human CE literature. Previous work has demonstrated their importance in colitis-induced colorectal cancer and inflammatory bowel disease, both chronic inflammatory conditions in humans (Perera *et al.* 2018, Friedrich *et al.* 2021). Both a decrease in miR-223 and subsequent increase in IL-1B and inflammasome activity are depicted as key players in the progression of these chronic conditions (Mao *et al.* 2018, Dosh *et al.* 2019). A similar pattern is seen in SCE in cows (Zhao *et al.* 2018). It is possible that the cellular pathways contributing to inflammation in the bovine endometrium are similar in women with CE. Indeed, Di Pietro *et al.* (2018) have already demonstrated differential expression of MiR-27a-3p and miR-124-3p in women with CE suggesting miRNA-mediated inflammatory pathways play a role in the pathogenesis of human CE. The interplay between miRNAs and markers of chronic inflammation may therefore act as both proxy markers for diagnosis of CE but may also point to underlying mechanistic pathways of disease.

The diagnosis of SCE in cows relies on identification of a high proportion of PMN cells within the uterus as determined by the relationship between PMN numbers and subfertility, or poor reproductive performance. Our thematic analysis identified poor reproductive performance as being associated with SCE in cows. In particular, cows which require more inseminations to conceive have a much higher prevalence of SCE than their herdmates which conceive upon their first insemination. This draws parallels with the impact of CE on human fertility (Buzzaccarini *et al.* 2020).

It is imperative to acknowledge certain limitations pertaining to the endometrial biology of the uterus in both the human as well as the bovine species. The endometrial shedding in women occurs cyclically at approximately 28-day intervals during menstruation (menstrual cycle). Conversely, reproductive cyclicity in cows manifests itself at approximately 21-day intervals, with the clinical expression being oestrus or heat, denoted as the oestrous cycle. Notably, cows do not undergo cyclic endometrial shedding, and the concept of menstruation is absent in this species. Furthermore, SCE in cows is predominantly observed in postpartum individuals facing challenges in adapting to the metabolic demands associated with heightened milk production. Metabolic stress, characterised by low glucose levels and elevated NEFAs, contributes to compromised innate immune function. This compromise includes the impaired ability of neutrophils to resolve inflammation and evacuate the uterine cavity, causing persistent endometrial inflammation in form of SCE. In contrast, in humans, CE is not inherently linked to the postpartum period or metabolic distress. The absence of such associations while underscoring points of mechanistic divergence also presents intriguing prospects for further research on CE among humans.

The present study is the first systematic review to explore diagnostic techniques and pathophysiology of SCE in cattle, using a thematic analysis, in order to highlight new areas of research for human CE. It is limited by the lack of direct comparisons between the condition in the two species to date.

In conclusion, our thematic systemic analysis of the literature on SCE in dairy cows has identified several areas that may be present within human CE yet remain underexplored. This offers the potential for new avenues of future research into human CE, and the possibility of novel, less invasive diagnostics and therapeutics for this disease.

## Supplementary Materials

Supplementary Material 1

Supplementary Material 2

## Declaration of interest

The authors declare that there is no conflict of interest that could be perceived as prejudicing the impartiality of the study reported.

## Funding

This research did not receive any specific grant from any funding agency in the public, commercial or not-for-profit sector. JO and NB’s time was funded by NIHR/MRC EME Project: 17/60/2. The open access fee for this article was funded by Tommy's.

## Author contribution statement

JO and SQ conceived of the project. KK and JO undertook the search, screening, data collection and data analysis. Initial manuscript preparation was conducted by KK and NB. KK, NB, EW, OBP, ST, SQ and JO reviewed and critically revised the manuscript.
