# Supplementary material for: Lessons learned from bovine subclinical endometritis: a systematic review exploring its potential relevance to chronic endometritis in women
Source: Reprod Fertil. 2024 Jun 5;5(2):e230035. doi: 10.1530/RAF-23-0035 (PMC11227093; doi:10.1530/RAF-23-0035)
Supplement: Supplementary Material 1 [file supplementary_material_1.pdf]

Supplementary Material 1: Search Strategy

A bespoke search strategy has been developed to ensure adequate identification of studies. This is outlined below:

The following MESH Headings will be used:

Endometritis

Reproductive Health

Female Infertility

Cow

Bovine

In addition, the following key words will be utilised: reproduct*, chronic endometritis, Cow*

The following electronic databases will be searched: Scopus, Embase, Medline, CINAHL
