# Supplementary material for: Lessons learned from bovine subclinical endometritis: a systematic review exploring its potential relevance to chronic endometritis in women
Source: Reprod Fertil. 2024 Jun 5;5(2):e230035. doi: 10.1530/RAF-23-0035 (PMC11227093; doi:10.1530/RAF-23-0035)
Supplement: Supplementary Material 2 [file supplementary_material_2.pdf]

Supplementary Material 2:

Supplementary Table 1: Extraction tool used to extract data from included papers.

Trial ID
Author
Year
Title
Journal
Country
Setting- Commercial (1)/ Domestic (2)
Sample Size
Study Period
No. of centres
Endometritis Definition used
Outcomes assessed
Pathophysiology (1)/ Diagnosis (2) paper
Relevant numbers
Highlighted Themes
